# 25-hydroxyvitamin D serum levels in patients with high risk resected melanoma treated in an adjuvant bevacizumab trial

**DOI:** 10.1038/s41416-018-0179-6

**Published:** 2018-07-23

**Authors:** Astrid Lipplaa, Ricardo Fernandes, Andrea Marshall, Paul Lorigan, Janet Dunn, Kevin A. Myers, Emily Barker, Julia Newton-Bishop, Mark R. Middleton, Pippa G. Corrie

**Affiliations:** 1 Department of Oncology, University of Oxford, CB2 0QQ Oxford, United Kingdom; 2 Warwick Clinical Trials Unit, University of Warwick, CV4 7AL Coventry, United Kingdom; 3 University of Manchester and Christie NHS Foundation Trust, M20 4BX Manchester, United Kingdom; 4 Experimental Cancer Medicine Centre, OX3 7DQ Oxford, United Kingdom; 5 Cambridge Cancer Trials Centre, Cambridge University Hospitals NHS Foundation Trust, CB2 0QQ Cambridge, United Kingdom; 6 Section of Epidemiology and Biostatistics, Leeds Institute of Cancer and Pathology, University of Leeds, LS9 7TF Leeds, United Kingdom; 7 NIHR Biomedical Research Centre, OX3 7LE Oxford, United Kingdom; 8 Cambridge Cancer Centre, Cambridge University Hospitals NHS Foundation Trust, CB2 0QQ Cambridge, United Kingdom; 9 Present Address: Leiden University Medical Center, Leiden, The Netherlands; 10 Present Address: Division of Medical Oncology, Department of Oncology, Schulich School of Medicine and Dentistry, Western University, London, ON, Canada

**Keywords:** Melanoma, Melanoma

## Abstract

**Background:**

Studies evaluating a relationship of vitamin D in patients with primary melanoma have consistently identified an inverse correlation with Breslow thickness, but an inconsistent impact on survival. Vitamin D in later stages of melanoma has been less studied.

**Methods:**

Vitamin D was measured in serum from 341 patients with resected stage IIB–IIIC melanoma recruited to the AVAST-M adjuvant melanoma randomised trial, collected prior to randomisation, then at 3 and 12 months. Vitamin D levels were compared with patient demographics, known melanoma prognostic factors, disease-free interval (DFI) and overall survival (OS).

**Results:**

A total of 73% patients had stage III melanoma, 32% were enroled (and therefore tested) >1 year after primary melanoma diagnosis. Median pre-randomisation vitamin D level was 56.5 (range 12.6–189.0 nmol/L). Vitamin D levels did not significantly vary over 12 months (*p* = 0.24). Individual pre-randomisation vitamin D levels did not differ significantly for Breslow thickness, tumour ulceration, or disease stage. Neither did pre-randomisation vitamin D predict for DFI (HR = 0.98 per 10 nmol/L increase; 95% confidence interval (CI) 0.93–1.04, *p* = 0.59) or OS (HR = 0.96 per 10 nmol/L increase, 95% CI 0.90–1.03, *p* = 0.31). For stage II patients, DFI improved with higher pre-randomisation vitamin D levels for those on bevacizumab (HR = 0.74 per 10 nmol nmol/L increase; 95% CI 0.56–0.97), but not for the observation arm (HR = 1.07 per 10 nmol/L increase; 95% CI 0.85–1.34).

**Conclusions:**

In this stage II/III melanoma cohort, vitamin D did not correlate with known prognostic markers, nor predict for DFI or OS, but there was some evidence of benefit for patients with stage II disease treated with bevacizumab.

## Introduction

Vitamin D is a pro-hormone primarily responsible for maintaining calcium and phosphate homoeostasis in the body.^1,2^ Its physiological functions include regulation of growth and differentiation in a wide variety of normal and malignant cells.^3^ The hormonally inactive pre-vitamin D is obtained from synthesis in the skin on exposure to sunlight and to some degree through diet and supplements. Two hydroxylation steps in the liver and kidney transform pre-vitamin D to the circulating form, 25-hydroxyvitamin D (25[OH]D, or vitamin D), and ultimately the active form, 1,25-hydroxyvitamin D (1,25[OH]D, or calcitriol).^4^

Large epidemiological studies have shown that serum vitamin D is inversely related to incidence and mortality of several malignancies.^5^ There are reported complex effects of vitamin D signalling through the vitamin D receptor (VDR) and indeed by non-genomic effects, as reviewed by Deeb et al.^6^ There are reported effects of vitamin D on cancer cells themselves and via effects on inflammation, T-cell function^7^ and the vasculature.^8^ Specifically, in vitro and in vivo studies have demonstrated that 1,25(OH)D exerts anti-proliferative and pro-apoptotic effects on different cancer cell lines.^9–11^ In melanoma, vitamin D has been shown to diminish cell adhesion, migration and growth of melanoma cells in vitro, as well as induce apoptosis.

The relationship between serum vitamin D levels and both melanoma occurrence and prognosis has been investigated in a number of studies.^12–18^ A meta-analysis^14^ identified that vitamin D levels at the time of diagnosis were inversely related to primary melanoma Breslow thickness, a well-established prognostic marker. There was no independent relationship between vitamin D and risk of melanoma recurrence, or survival. However, the meta-analysis predates several large-scale vitamin D prospective cohort studies and the three largest of these performed to date involving over 1000 participants each have independently reported an association between vitamin D and melanoma relapse and/or survival.^12,15,17^ Other reports have suggested a relationship between VDR gene polymorphisms and melanoma risk^19^ as well as disease-specific survival,^20,21^ although these data are not conclusive.

Few therapeutic trials of vitamin D supplements have been undertaken to date and have, so far, not shown any reduction in melanoma recurrence or incidence. The Women’s Health Initiative randomised controlled trial found no change in melanoma incidence in women who had taken supplements for an average of 7 years.^22^ Results are pending for the Mel-D trial investigating adjuvant treatment with vitamin D in patients with resected stage IIB–IIIB melanoma.^23,24^

Most of the data correlating vitamin D levels with melanoma prognosis come from studies performed in patients who recently had a primary tumour resected, most of whom had a high chance of cure from surgery. Studies, to date, have included relatively few patients with resected locoregional melanoma, who are at much higher risk of relapse and death from their disease. We therefore measured serum vitamin D in a cohort of patients from the UK AVAST-M study, which evaluated the role of the VEGF inhibitor, bevacizumab, as adjuvant treatment for patients with resected AJCC stage IIB–IIIC melanoma: a population whose median 5 year overall survival was 64%.^25,26^

## Materials and methods

### Study design and patients

The AVAST-M trial was an open-label, randomised controlled phase 3 trial that enroled 1343 patients between 18 July 2007 and 29 March 2012 at 48 UK centres. Eligible patients were at least 16 years old, with histological confirmation of completely resected AJCC (7^th^ edition) stage IIB-C and IIIA-C melanoma. Patients were randomly assigned to treatment with bevacizumab, 7.5 mg/kg every 3 weeks for 1 year, or observation. Randomisation occurred within 12 weeks of surgical resection. Outcomes included overall survival (OS) and disease-free interval (DFI). Further details and results of the trial are reported elsewhere.^25,26^ We identified patients enroled in the AVAST-M trial with suitable serum aliquots collected after their melanoma surgery and within 28 days of trial randomisation, then at 3 and 12 months (unless relapse occurred before 12 months) for vitamin D testing. The trial protocol required use of concomitant medications, including supplements and complementary therapies, to be recorded at each clinic visit.

### Vitamin D measurement

We measured serum 25(OH)D (vitamin D) using liquid chromatography and mass spectrometry on samples collected pre-randomisation, then 3 and 12 months after randomisation. Vitamin D results were reported in nmol/L.

### Statistical analysis

A power calculation was performed which suggested that a minimum sample size of 340 patients would be sufficient to detect a standardised difference of 0.3 in vitamin D levels between patient characteristic groups with at least 80% power and a 5% significance level. It was also sufficient to detect a hazard ratio of 0.65 for OS.

Box and whisker plots were constructed displaying the median, interquartile range and ranges for the pre-randomisation vitamin D levels depending on the month of measurement. A generalised linear model was used to assess if the pre-randomisation continuous vitamin D levels differed across season, primary melanoma Breslow thickness and presence or absence of ulceration, disease stage at randomisation, time between diagnosis and randomisation, as well as trial arm, after adjustment for pre-randomisation covariates of age, gender and body mass index (BMI), these being factors known to affect vitamin D levels. A log transformation was used to make the assumption of normality more appropriate. DFI was calculated as the time from randomisation until the first tumour recurrence or date of death due to melanoma. OS was calculated from randomisation until the date of death from any cause. A Cox proportional hazards model was fitted to assess the association of vitamin D on OS and DFI after adjustment for pre-randomisation covariates and trial arm allocation. Similar analyses were undertaken for the primary melanoma (stage II) and locoregional disease (stage III) subgroups, although the subgroup sizes were comparatively small: 90 stage II and 251 stage III patients made up the total cohort studied here.

For the patients with vitamin D measured over three time-points, individual profile plots were generated to identify any patterns in changing vitamin D levels over time. Mixed effects models were used to assess the effects of vitamin D levels over time, accounting for the random variability between patients and the repeated measures. These models were also adjusted for the pre-randomisation covariates of age, gender, BMI and the season when the measurement was taken. Mixed effects models were also used to assess whether vitamin D levels over time differed according to primary melanoma Breslow thickness or ulceration at the time of initial diagnosis or disease stage at randomisation, after adjustment for pre-randomisation covariates and season.

### Role of the funding source

The sponsor and funder of the study had no role in study design, data collection, data analysis, data interpretation or writing of this report.

## Results

### Evaluation of pre-randomisation vitamin D levels

A total of 341 patients from the AVAST-M trial had pre-randomisation serum available for vitamin D measurements. The demographic and disease characteristics of this sub-group were similar to those for the whole trial population (Table 1 and Corrie et al.,^25^). A total of 251 (73%) patients had stage III melanoma, 137 (40%) had an ulcerated primary tumour and 120 were staged with sentinel lymph node biopsy. Pre-randomisation vitamin D was measured in blood collected at trial enrolment, but the primary melanoma Breslow thickness and presence of ulceration were determined at initial diagnosis, which may have been several years prior to AVAST-M trial entry and randomisation in those patients who entered the trial after resection of stage III melanoma: median time from diagnosis to randomisation was 0.47 years, range 0–22.6 years (Table 1). Approximately one third (108, 32%) of patients were enroled >1 year after primary melanoma diagnosis.

**Table 1:** Patient and melanoma disease characteristics

Characteristic	All AVAST-M trial patients *N *(%)	Vitamin D cohort *N* (%)	Bevacizumab *N* (%)	Observation *N* (%)	Stage II disease *N* (%)	Stage III disease *N* (%)
	1343	341	171	170	90	251
*Gender*						
Male	753 (56)	186 (55)	88 (51)	98 (58)	58 (64)	128 (51)
Female	590 (44)	155 (45)	83 (49)	72 (42)	32 (36)	123 (49)
Age in years, median [range]	56 (18–88)	55 (19–86)	55 (19–80)	56 (19–86)	64 (23–86)	53 (19–80)
BMI kg/m^2^, median [range]	27.6 [15.7–68.6]	26.8 [15.8–57.6]	27.1 [15.8–57.6]	26.5 [19.4–46.2]	28.4 [19.7–40.1]	26.4 [15.8–57.6]
Time from initial melanoma diagnosis to randomisation in years, median [range]	0.46 [0–29.3]	0.47 [0–22.6]	0.45 [0.12–18.3]	0.48 [0–22.6]	0.32 [0.13–0.45]	0.64 [0–22.6]
<1	912 (68)	233 (68)	120 (70)	113 (67)	90 (100)	143 (57)
1–2	130 (10)	40 (12)	21 (12)	19 (11)	0	40 (16)
>2	301 (22)	68 (20)	30 (18)	38 (22)	0	68 (28)
*Primary melanoma Breslow thickness at diagnosis, in mm*						
≤2	399 (30)	103 (30)	51 (30)	52 (31)	0	103 (30)
>2–4	405 (30)	107 (31)	54 (31)	53 (31)	24 (27)	83 (33)
>4	438 (33)	111 (33)	56 (33)	55 (32)	66 (73)	45 (18)
Unknown	101 (7)	20 (6)	10 (6)	10 (6)	0	20 (6)
*Primary melanoma ulceration at diagnosis*						
Present	518 (39)	137 (40)	70 (41)	67 (39)	67 (74)	70 (28)
Absent	633 (47)	151 (44)	68 (40)	83 (49)	20 (22)	131 (52)
Unknown	192 (14)	53 (16)	33 (19)	20 (12)	3 (3)	50 (20)
*Disease stage at trial entry*						
II	364 (27)	90 (27)	48 (28)	42 (24)		
IIIA	195 (15)	52 (15)	27 (16)	25 (15)		
IIIB	495 (37)	113 (33)	54 (32)	59 (35)		
IIIC	289 (21)	86 (25)	42 (24)	44 (26)		

**Fig. 1:**
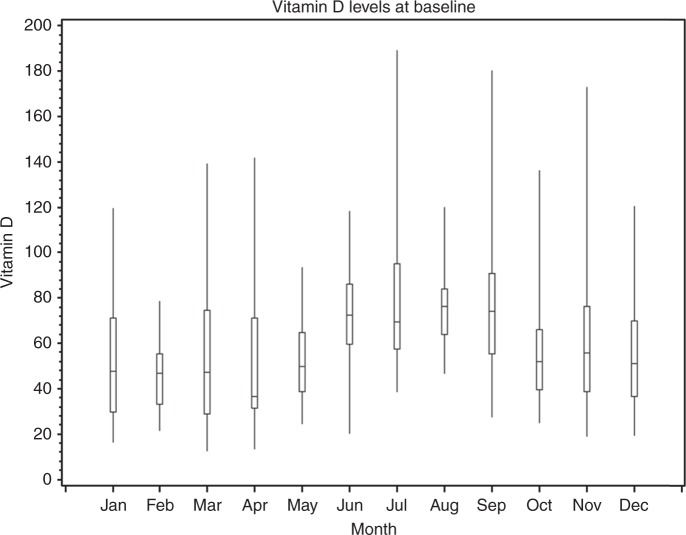
Plots of pre-randomisation vitamin D levels against the month the sample was taken. Boxes represent interquartile range of vitamin D (nmol/L), whiskers represent range

The median pre-randomisation vitamin D level was 56.5 nmol/L (interquartile range 38.5–76.5 nmol/L; range 12.6–189.0 nmol/L) (Table 2). Pre-randomisation levels were very similar for both resected stage II and stage III subgroups. Twenty-four patients had vitamin D levels <25 nmol nmol/L and an additional 9 patients had vitamin D levels <45  nmol/L during the summer months of July–September. Pre-randomisation vitamin D levels varied according to the month in which the sample was taken. In a generalised linear regression model after adjustment for age, gender and BMI, vitamin D levels varied depending on the season (*p* < 0.001), with higher levels from July to September (Table 2; Fig. 1).

**Table 2:** Pre-randomisation vitamin D levels for the season when sample was taken and association with specific melanoma disease characteristics

		Pre-randomisation vitamin D levels in nmol/L
	*N*	Median [interquartile range]
All patients	341	56.5 [38.6–76.5]
*Timing of sample (season)*		
January–March	96	47.1 [30.0–69.0]
April–June	77	54.3 [36.3–75.3]
July–September	77	73.3 [57.5–90.7]
October–December	91	52.2 [38.0–68.3]
*Primary melanoma Breslow thickness at diagnosis in mm*		
≤2	103	54.5 [36.2–72.6]
>2-4	107	56.9 [39.1–82.3]
>4	111	56.9 [43.1–75.7]
Unknown	20	56.3 [38.9–82.4]
*Primary melanoma ulceration*		
Present	137	54.9 [41.0–76.7]
Absent	151	60.1 [36.9–77.2]
Unknown	53	53.3 [39.1–71.2]
*Disease stage at trial entry*		
II	90	55.6 [42.3–76.6]
III	251	56.6 [37.7–76.5]
*Time from initial melanoma diagnosis to randomisation in years*		
<1	233	56.5 [40.0–76.6]
1–2	40	54.3 [36.3–75.9]
>2	68	57.2 [37.4–71.6]
*Trial arm*		
Bevacizumab	171	52.6 [37.5–76.7]
Observation	170	59.8 [39.8–76.5]

Twelve patients reported taking vitamin D supplements pre-randomisation and their median vitamin D level was 67.9 nmol/L (range 52.2–99.5). These were not the patients with the highest vitamin D concentrations. During the subsequent 12 month period, a further 23 patients started taking vitamin D supplements and their median pre-randomisation vitamin D level was 50.0 nmol/L (range 19.5–119.8); only 1 patient had a vitamin D level <25 nmol/L. For this combined cohort of 35 patients taking vitamin D supplements, their vitamin D levels at 12 months (median 61.6 nmol/L (range 26.8–123.7) were not significantly higher than for those patients who did not (median 51.2 nmol/L (range 10.3–166.2; *p* = 0.10 unadjusted, *p* = 0.07 adjusted for age, gender, BMI and season).

In a generalised linear regression model, pre-randomisation vitamin D levels did not differ significantly for primary melanoma Breslow thickness (*p* = 0.72), ulceration (*p* = 0.33), disease stage at trial entry (*p* = 0.25), time between diagnosis (*p* = 0.23) and randomisation, or trial arm (*p* = 0.84), after adjustment for age, gender, BMI, and season (Table 3). The findings were not significantly different when analysed separately by disease stage II and III subgroups.

**Table 3:** Associations of melanoma disease characteristics and pre-randomisation vitamin D levels

Characteristic	Total *p*-value	Disease stage II *p*-value	Disease stage III *p*-value
Number of patients	341	90	251
Season	<0.001	0.02	<0.001
Primary melanoma Breslow thickness at diagnosis, in mm	0.72	0.59	0.78
Primary melanoma ulceration at diagnosis	0.33	0.83	0.23
Time from initial melanoma diagnosis to randomisation in years, median [range]	0.23	^a^	0.30
Trial arm	0.84	0.64	0.96

*P*-values reported from a GLM model for pre-randomisation vitamin D levels and after adjustment for age gender, BMI, and season.

^a^Cannot calculate for stage II as all were within the same group

Of the 341 patients in this analysis, 120 (35%) have died; 109 (91%) from metastatic melanoma. The median follow-up for the 221 surviving patients was 6 years. Pre-randomisation vitamin D level did not predict for OS (HR = 0.96 per 10 nmol/L increase (95% confidence interval (CI) 0.90–1.03); *p* = 0.31) after adjustment for age, gender, BMI, season, and trial arm. A total of 169 (50%) had relapsed either locally or distantly. Pre-randomisation vitamin D level did not predict for DFI (HR = 0.98 per 10 nmol/L increase (95% CI 0.93–1.04); *p* = 0.59) after adjustment for age, gender, BMI, season, and trial arm. The 24 patients classified as being vitamin D deficient (<25 nmol/L^27^) did not have significantly worse outcomes in terms of OS (*p* = 0.42) or DFI (*p* = 0.78), compared with those patients with higher vitamin D levels.

There was no significant interaction between trial arm and vitamin D levels for OS (*p* = 0.70) or DFI (*p* = 0.29). Similar results within the disease stage II and III subgroups were seen, with one exception: for the stage II subgroup, there was a significant interaction between vitamin D and trial arm for DFI (*p* = 0.03), which was not seen for the stage III subgroup or the combined cohort (Table 4). For stage II patients randomised to bevacizumab, DFI improved with higher pre-randomisation vitamin D levels (HR = 0.74 per 10 nmol/L increase; 95% CI 0.56–0.97). This was not the case for the observation arm (HR = 1.07 per 10 nmol/L increase; 95% CI 0.85–1.34).

**Table 4:** Association of vitamin D levels with disease-free interval and overall survival

Characteristic	Vitamin D cohort	Stage II subgroup	Stage III subgroup
Number of patients	341	90	251
*Overall survival*			
Vitamin D	HR = 0.96 (95% CI 0.90–1.03); *p* = 0.31	HR = 0.89 (95% CI 0.74–1.08); *p* = 0.24	HR = 0.98 (95% CI 0.91–1.06); *p* = 0.61
Vitamin D and trial arm interaction	*P* = 0.70	*P* = 0.58	*P* = 0.59
*Disease-free interval*			
Vitamin D	HR = 0.98 (95% CI 0.93–1.04); *p* = 0.59	HR = 0.91 (95% CI 0.77–1.08); *p* = 0.27	HR > 0.99 (95% CI 0.94–1.06); *p* = 0.98
Vitamin D and trial arm interaction	*P* = 0.29	*p* = 0.03	*P* = 0.74
Vitamin D for Bevacizumab arm only		HR = 0.74 (95% CI 0.56–0.97)	
Vitamin D for Observation arm only		HR = 1.07 (95% CI 0.85–1.34)	

^a^All hazard ratios for vitamin D are for a 10 nmol/L increase in vitamin D level and obtained after adjustment for age gender, BMI, and season.

### Vitamin D levels over time

A total of 175 (51%) of the 341 patients had vitamin D measurements available at all three time points: pre-randomisation, 3 and 12 months after randomisation. There was no consistent pattern for any changes in vitamin D levels over time. For individuals, vitamin D varied over the three time-points by a median 20.4 nmol/L (interquartile range 11.1–32.0, range 0.4–116.3). The median vitamin D level at each time-point was similar (Supplementary Table 1).

Pre-randomisation vitamin D levels varied according to the season of the year, with higher levels during July–September and similar patterns were seen in samples taken at 3 and 12 months. After adjustment for age, gender and BMI, the seasonal variation was statistically significant (*p* < 0.001), but no variation between the three time-points was observed (*p* = 0.24). There was also no association between vitamin D levels over time and primary melanoma Breslow thickness (*p* = 0.30), ulceration (*p* = 0.41) or disease stage at trial entry (*p* = 0.17, Supplementary Table 1).

## Discussion

We performed an exploratory study to measure vitamin D in a cohort of melanoma patients who took part in the AVAST-M trial, an adjuvant trial investigating the use of bevacizumab in patients with melanoma at a high risk of recurrence. At the time of initiating AVAST-M, angiogenesis inhibition was a relevant target for cancer adjuvant therapy, including melanoma, based on early signals of efficacy in metastatic disease.^28^ Since there was no known pharmacological interaction between bevacizumab and vitamin D, we made use of serial blood samples collected from patients in both the observation and treatment arms and explored relationships of vitamin D with disease characteristics and outcomes after surgery, including disease recurrence and survival. In comparison with primary melanoma populations in whom vitamin D has been studied to date, the sample size of our study was relatively small: 341 patients had available pre-randomisation vitamin D measurements and half of these had subsequent measurements performed during the first year of follow-up. Even so, consistent with the previously reported literature,^29–31^ we found strong evidence that vitamin D varied seasonally, with the highest serum levels from July to September.

In contrast to the larger scale primary melanoma studies, which have reported an association between vitamin D and melanoma relapse and/or survival (Table 5), our study in patients with predominantly later stage disease showed no evidence that vitamin D was an independent prognostic marker after surgical resection. Our cohort comprised 90 resected stage II and 251 resected stage III patients. Within these smaller subgroups, the findings were no different to the whole population.

**Table 5:** Overview of studies investigating the relationship between vitamin D levels and melanoma outcomes and/or prognostic factors

Study	Sample size	Population		Melanoma outcomes	Prognostic factors
		Stage	Breslow thickness		
Newton-Bishop et al.^12^	1130	I–IIIA	30% >2 mm	Per 20 nmol/L increase vitamin D level: HR relapse: 0.79 (95% CI, 0.64–0.96) HR overall death: 0.83 (95% CI, 0.68–1.02)	Vitamin D level inversely associated with: Breslow thickness (*p* = 0.002)
Nurnberg et al.^42^	205	0–IV	—	Trend towards earlier distant metastatic disease with vitamin D level <25 nmol/L versus >50 nmol/L: 24.4 versus 29.5 months (*p* = 0.641)	Vitamin D level inversely associated with: Breslow thickness (NS, *p* = 0.078); Stage (*p* = 0.006)
Gambichler et al.^13^	764	0–IV (59% stage ≤I)	—	—	Vitamin D level inversely associated with: Breslow thickness (*p* = 0.028); Stage (*p* = 0.036) No association between vitamin D level and ulceration status
Ogbah et al.^43^	81	—	68% ≤1 mm	—	No association between vitamin D level and Breslow thickness
Saiag et al.^15^	1171	I–IV (55% stage ≤I)	31% >2 mm	Vitamin D level at pre-randomisation not associated with prognosis (relapse/death). Vitamin D level increase (>4.60 nmol/L/year) and decrease (<5.25 nmol/L/year) associated with increased risk of relapse	Vitamin D level inversely associated with: Breslow thickness (*p* < 0.001); Stage (*p* < 0.001); Ulceration status (*p* < 0.001)
Wyatt et al.^16^	100	I–II	17% >0.75 mm		Vitamin D level inversely associated with: Breslow thickness (*p* = 0.04) No association with Clark level, presence of mitoses Highest levels of Vitamin D not associated with more favourable prognosis
Fang et al.^17^	1042	I–IV (66% stage I–II)	29% >2 mm	Vitamin D level inversely associated with: OS (*p* < 0.001); MSS (*p* = 0.0025); DFS (*p* = 0.047)	Vitamin D level inversely associated with: Breslow thickness (*p* < 0.001); Stage (P < 0.0024); Ulceration status (*p* = 0.0105)
Timerman et al.^18^	252	0–IV (18% stage ≤I)	25% <1 mm 22% >4 mm	Vitamin D level <20 nmol/L associated with increased risk of melanoma related death, HR 1.93 (95% CI, 1.15–3.22) Vitamin D level decrease, and increase <20 nmol/L, associated with increased risk of melanoma related death, HR 4.68 (95% CI, 1.05–20.88)	Vitamin D level inversely associated with: Stage (*p* = 0.01) No association between vitamin D level and Breslow thickness or ulceration
AVAST-M cohort	341	IIB–IIIC	64% >2 mm	Vitamin D level not associated with prognosis (relapse/death)	Vitamin D level not associated with Breslow thickness, stage or ulceration status

*DFS* disease-free survival, *OS* overall survival, *HR* hazard ratio, *CI* confidence interval, *MSS* melanoma specific survival, *NS* non-significant

Our findings could still be consistent with an anti-cancer effect of vitamin D in the early stages of carcinogenesis, which is lost once the cancer progresses. The genomic effects of vitamin D are mediated when 1,25:dihydroxyvitamin D is bound to a heterodimeric receptor formed from the retinoid X receptor (RXR) and the vitamin D receptor (VDR).^32^ Vitamin D is also known to have non-genomic effects.^6^ The antiproliferative effects of vitamin D on normal and cancer cells in vitro are well described^10^ and there is good evidence that some of those effects at least are mediated by the induction of E-cadherin.^33^ Evidence that the majority of the antiproliferative effects are genomically mediated by VDR has been reported after the restoration of growth inhibitory effects of vitamin D in VDR null murine cells, engineered to stably express VDR.^34^ The observation of a correlation between vitamin D levels and the thickness of primary melanoma at diagnosis in previously published studies (Table 5) is consistent with the hypothesis that low vitamin D may contribute to the growth of primary melanoma. It is known however that there is progressive loss of expression of VDR with melanoma progression^35^ and it is therefore likely that any protective effect of vitamin D on cell proliferation will be diminished or lost in more advanced tumours.

Vitamin D is known to have complex effects on cells and of particular concern for cancer is a wealth of literature suggesting that vitamin D mediates an immunosuppressive effect in humans.^36,37^ We have therefore expressed concern previously that there might be a narrow therapeutic window for a putative beneficial effect of vitamin D in melanoma^38^ and the view that a conservative approach to supplementation should be adopted in order to avoid higher serum levels to avoid potential harm.^39^ A transcriptomic study^40^ reported evidence that higher vitamin D levels in melanoma patients were associated with less proliferative tumour phenotypes and a greater likelihood of a stronger immune gene signature. This is reassuring from a safety point of view, but it remains of concern that in the presence of loss of expression of VDR, higher vitamin D levels might be associated with adverse outcomes for poorer prognosis melanoma patients rather than having a protective effect. In our study, we saw no evidence that higher vitamin D levels were associated with a deleterious effect on survival, although the sample size was quite small.

There is an obvious major difference in our approach compared with previously published series. Compared with most other studies, which measured vitamin D after resection of primary melanoma, in our cohort blood samples were taken predominantly from patients after resection of regional lymph nodes, representing relapse of disease sometimes years after the initial diagnosis and treatment of their primary melanoma. For many of our patients there was ample time for their health behaviour to change between their primary diagnosis and the melanoma event that occasioned trial entry. Vitamin D levels might therefore reflect overall health and modified sun exposure or dietary intake, instead of being a causal factor in melanoma recurrence, while the vitamin D level measured at the time of trial entry may not reflect that at the time of primary melanoma diagnosis when tumour thickness was determined.

Vitamin D levels were higher overall in our cohort compared to the previous studies performed by Newton-Bishop et al.^12^ and Saiag et al.^15^: only 7% of our patient cohort would have been classified as vitamin D deficient using a nationally agreed definition of <25 nmol/L.^27^ Ten percent of the patients we studied were taking vitamin D supplements. Although only 1 of these patients had pre-randomisation vitamin D level <25 nmol/L, taking supplements did not ensure the highest vitamin D levels.

Of note, the only significant correlation with outcomes we identified was that higher pre-randomisation vitamin D levels predicted for longer DFI in the bevacizumab-treated stage II patients only. The relevance of this finding is not clear and given the very small sample size, it could be a chance finding. On the other hand, current literature suggests that vitamin D has both anti-angiogenic and pro-angiogenic effects^8^ and the fact that this outcome was identified only in the stage II subgroup may be evidence of vitamin D exerting an influential effect in the earlier stages of tumour invasion. In the context of metastatic colorectal cancer, a randomised phase II trial recently reported improved progression-free survival when patients treated with standard bevacizumab + mFOLFOX6 systemic therapy received high versus low vitamin D supplementation.^41^ A positive interaction between vitamin D and bevacizumab with therapeutic potential cannot, at this stage, be discounted.

In conclusion, as previously reported, we have shown seasonal variation in vitamin D levels. In contrast to published studies previously describing a relationship between vitamin D and primary melanoma thickness, in this study of 341 patients with later stage resected stage II–III melanoma recruited to the AVAST-M trial, there was no correlation between vitamin D and primary melanoma Breslow thickness, ulceration or disease stage. Overall, Vitamin D levels did not predict for subsequent relapse or survival. These findings contrast with stronger reported associations with melanoma stage at diagnosis and may reflect changes in biological effects of vitamin D at later stages of the disease. The observation of a better DFI in bevacizumab-treated stage II patients with higher levels of vitamin D warrants further exploration before justifying future exploitation.

### Data availability

The authors confirm that the data supporting the findings of this study are available within the article and its supplementary materials.

## Electronic supplementary material

Supplementary Table 1
