# Supplementary material for: 25-hydroxyvitamin D serum levels in patients with high risk resected melanoma treated in an adjuvant bevacizumab trial
Source: Br J Cancer. 2018 Jul 23;119(7):793–800. doi: 10.1038/s41416-018-0179-6 (PMC6189120; doi:10.1038/s41416-018-0179-6)
Supplement: Supplementary file 1 — Supplementary Table 1 [file 41416_2018_179_MOESM1_ESM.docx]

**Supplementary Table 1:** Summary of the vitamin D levels (in nmol/L) at each sampling time-point for the season when sample was taken and across tumour characteristics

|  | Pre-randomisation | 3 months | 12 months |
| --- | --- | --- | --- |
|  | Median  [interquartile range] | Median  [interquartile range] | Median  [interquartile range] |
| Vitamin D levels (nmol/L) | 53.0 [37.4 – 72.5] | 53.9 [37.7 – 75.3] | 53.2 [34.0 – 78.4] |
| Timing of sample (Season) |  |  |  |
| January-March | 45.6 [29.9-68.3] | 42.4 [31.5-60.3] | 37.3 [29.0-61.3] |
| April-June | 51.3 [37.3-73.5] | 54.9 [39.5-78.1] | 52.2 [32.0-66.6] |
| July-September | 69.9 [56.6-80.8] | 66.1 [46.1-88.0] | 78.4 [55.0-97.8] |
| October-December | 50.0 [35.9-68.0] | 55.3 [36.9-74.2] | 56.0 [40.8-76.0] |
| Breslow Thickness at diagnosis |  |  |  |
| ≤ 2mm | 51.5 [29.9-68.0] | 51.5 [36.9-70.9] | 53.5 [34.5-78.7] |
| > 2-4mm | 54.8 [39.1-77.4] | 62.8 [37.8-86.1] | 57.1 [35.4-81.8] |
| > 4mm | 53.4 [41.8-75.1] | 55.2 [38.3-75.4] | 52.7 [34.0-76.0] |
| Unknown | 46.3 [34.0-61.8] | 52.9 [39.2-62.7] | 45.6 [32.6-58.9] |
| Ulceration at diagnosis |  |  |  |
| Present | 54.0 [39.8-78.1] | 60.1 [33.9-83.2] | 54.8 [3.8-78.7] |
| Absent | 53.6 [36.7-72.0] | 53.4 [39.1-74.6] | 54.9 [34.8-80.0] |
| Unknown | 43.5 [34.7-61.8] | 49.3 [38.0-64.7] | 45.7 [32.6-63.1] |
| Disease stage at randomisation |  |  |  |
| II | 53.4 [40.4-73.3] | 57.7 [38.6-75.7] | 52.1 [34.0-74.4] |
| III | 51.6 [36.7-71.3] | 53.0 [37.3-75.0] | 53.4 [34.5-78.7] |
